# Hospital Annual Meetings

**Published:** 1920-03-06

**Authors:** 


### Hospital Annual Meetings.

### King's College Hospital.

Viscount Hambleden presided last week at the Annual Court of Governors of King's College Hospital, which has been busy during the past year in reconstructing and re-organising after the occupation by the military. Expenditure in 1919 amounted to £102,500, £9,300 more than in 1918. The hospital experienced a deficiency last year of £43,000, which was met as to £540 by legacies—the smallest amount for many years—and as to £21,000 by donations received in response to special appeals, leaving an ultimate deficit of £21,359, which was borrowed. The debt on the hospital, notwithstanding the generosity of a donor in discharging the £40,000 borrowed in 1918 for paying the cost of the building, is now over £67,000, only £20,000 less than it was at the end of 1918. If within their means, out-patients are now asked to pay 6d. per attendance—a method whereby it is hoped to secure £4,000 annually. Arrangements have now been made at the hospital for middle-class paying patients. Five wards are at present closed through want of funds.

### Royal London Ophthalmic Hospital.

In-patients to the number of 2,555, a larger number than in any previous year, were cared for in 1919 by the Royal London Ophthalmic Hospital, the annual meeting of which was held last week. New in-patients during that period numbered 38,675, and their attendances 105,526. Over 4,660 L.C.C. school children, for whom 3,768 spectacles were prescribed, also received attention. Income in 1919 fell short of the expenditure by £4,665, and the institution's debt to its bankers is £5,000. Improvements to heating apparatus and the provision of steam-heated sterilisers have incurred a capital expenditure of £2,671. Patients' contributions last year amounted to £1,393—a small sum, says the Committee, when the number of persons benefited is realised. It is hoped to increase the range of contributors to the institution, it being felt that it is not right that the national work performed there should be supported only by some 1,500 people.

### City of London Maternity Hospital.

The financial position of the City of London Maternity Hospital, the annual meeting of which was held last week, is still a matter for grave concern, the indebtedness to the bankers being £7,200. However, the income last year amounted to £12,557, £3,752 more than in 1918, and exceeded the expenditure of £12,441 by nearly £146. During 1919 1,486 patients, of whom 1,403 were delivered, were admitted to the hospital, being 135 more than in 1918, and exceeding by 96 the highest number previously reached. The patients gave birth to 1,428 children, of whom 777 were boys and 649 girls. These figures constitute a record in the history of the hospital. The preponderance of male births is the greatest for thirty-four years. Twenty-three patients had twins, the highest figure on record, and fifty-five children were still-born, while five women and forty children died, being, in the case of the women, the lowest percentage since 1909. In the out-patient department 428 women were delivered in their own homes, as against 290 in 1918. Forty-eight medical practitioners and students attended the midwifery practice of the Institution last year, being 14 more than in 1918.

### Central London Ophthalmic Hospital.

Mr. Brittin Archer presided last week over the Annual Court of Governors of the Central London Ophthalmic Hospital, which in 1919 admitted 488 patients, against 386 in the previous year. New out-patients numbered 13,008, as compared with 12,436 in 1918, while 803 accident cases were treated. Major and minor operations to the numbers of 367 and 1,232 respectively were performed during the year under review. Donations in 1919 were £858 less than the year before, while expenditure increased by £628 from £4,242 to £4,870. The institution has benefited considerably by a legacy from the executors of Mrs. Selina Artiss, consisting of the residue of her estate, and amounting to £5,716, made up of many small investments, some of which it has already been found necessary to realise. The legacy enables the balance-sheet to show an excess of income over expenditure of £4,502. The Ministry of Pensions is still sending to the hospital for both in- and out-patient treatment many demobilised wounded soldiers and sailors suffering from eye trouble.

### St. Mark's Hospital.

St. Mark's Hospital, City Road, the Annual Meeting of which was held recently, cared for 646 in-patients last year, 558 of whom were discharged cured. The Committee reported that not only had the building site adjoining the institution been acquired through the untiring efforts of Mr. R. H. Martin, the Treasurer, and the gentlemen associated with him, but that a surplus amounting to £128 had been presented to the hospital for building purposes. The serious menace which threatened the hospital by the erection of a factory shutting out light and air so necessary to the health of the patients was now averted, and a valuable site had been acquired, and provision made for extension when need should arise. The Committee were anxious to remove the derelict building which still stood on the newly acquired site in order to have an open space, where the patients might lie. To do this would cost £400, and an opportunity was thus afforded for a generous donor to do real service towards the restoration to health of the patients. Income last year exceeded expenditure by nearly £100.

### St. Mary's Hospital, Plaistow.

Bishop Stevens presided over the Annual Court of Governors of St. Mary's Hospital for Women and Children, Plaistow, held last week. Although the daily average of in-patients treated last year showed a slight decrease as compared with 1918, out-patients, who numbered 8,050, were 2,015 more. Total expenditure for 1919 amounted to £6,731, £1,020 more than in 1918, and £1,081 greater than the income, which was £5,650. A letter was read from King Edward's Hospital Fund approving the plans for the rebuilding of the nurses' home and out-patient department, and offering no objection to the work being proceeded with. The Fund has already made grants totalling £5,500 for this purpose. The sum required for rebuilding is £60,000, £13,444 having already been collected, leaving a balance of £46,556. The King's Fund is prepared to recommend its Council to afford further help, since it recognises the urgency of the improvements, but that further financial assistance will be proportionate to some extent to the amount obtained as the result of the appeals to the public.

